# Simulated galactic cosmic ray exposure activates dose-dependent DNA repair response and down regulates glucosinolate pathways in arabidopsis seedlings

**DOI:** 10.3389/fpls.2023.1284529

**Published:** 2023-12-14

**Authors:** Anirudha R. Dixit, Alexander D. Meyers, Brian Richardson, Jeffrey T. Richards, Stephanie E. Richards, Srujana Neelam, Howard G. Levine, Mark J. Cameron, Ye Zhang

**Affiliations:** ^1^ AETOS Systems Inc., LASSO II Contract, Huntsville, AL, United States; ^2^ NASA Postdoctoral Program, John F. Kennedy Space Center, Merritt Island, FL, United States; ^3^ Case Western Reserve University, Cleveland, OH, United States; ^4^ Bionetics Corp., LASSO Contract, Merritt Island, FL, United States; ^5^ NASA John F. Kennedy Space Center, Kennedy Space Center, FL, United States

**Keywords:** galactic cosmic ray, space radiation, Arabidopsis, DNA damage response, glucosinolate pathway, transcriptomic analysis

## Abstract

Outside the protection of Earth’s magnetic field, organisms are constantly exposed to space radiation consisting of energetic protons and other heavier charged particles. With the goal of crewed Mars exploration, the production of fresh food during long duration space missions is critical for meeting astronauts’ nutritional and psychological needs. However, the biological effects of space radiation on plants have not been sufficiently investigated and characterized. To that end, 10-day-old *Arabidopsis* seedlings were exposed to simulated Galactic Cosmic Rays (GCR) and assessed for transcriptomic changes. The simulated GCR irradiation was carried out in the NASA Space Radiation Laboratory (NSRL) at Brookhaven National Lab (BNL). The exposures were conducted acutely for two dose points at 40 cGy or 80 cGy, with sequential delivery of proton, helium, oxygen, silicon, and iron ions. Control and irradiated seedlings were then harvested and preserved in RNAlater at 3 hrs post irradiation. Total RNA was isolated for transcriptomic analyses using RNAseq. The data revealed that the transcriptomic responses were dose-dependent, with significant upregulation of DNA repair pathways and downregulation of glucosinolate biosynthetic pathways. Glucosinolates are important for plant pathogen defense and for the taste of a plant, which are both relevant to growing plants for spaceflight. These findings fill in knowledge gaps of how plants respond to radiation in beyond-Earth environments.

## Introduction

Deep space radiation is composed primarily of solar particle events (SPEs) and galactic cosmic rays (GCR). Cosmic rays (GCR and SPEs) consist of approximately 85-90% protons, and 10-13% helium ions (alpha particles), with the remaining 1-2% consisting of high-atomic number and energy (HZE) nuclei particles and electrons (Townsend, 2005; Zhang et al., 2022). For future missions to the Moon or to Mars, astronauts and other biological organisms onboard these missions will be exposed constantly to GCR and occasionally to radiation from large SPEs. Life on Earth is well protected from these components of deep-space radiation for two reasons: (1) the Earth’s global magnetic field deflects energetic charged particles, and (2) the atmosphere interacts with them resulting in attenuated energy levels and lower energy secondary particles. Organisms in Low Earth Orbit (LEO) are still within Earth’s magnetic field and therefore partially shielded from radiation. Missions beyond LEO, however, will not have such protections. The GCR, SPEs, and secondary particles experienced during these missions will be more intense, and understanding the biological impacts is crucial to extend deep space exploration.

During long-term deep space missions, it is expected that seeds and plants growing in space will be exposed to 1-2 mSv/day GCR and approximately half this amount on planetary surfaces (Cucinotta and Durante, 2006; Zeitlin, 2013; Huff et al., 2016). This is much higher than the approximately 2.4 mSv/year natural radiation typically experienced on Earth. While the flux levels of GCR particles are very low, these high-linear energy transfer (LET) particles produce intense ionization as they pass through matter. Therefore, the average quality factor of radiation received on the Martian surface against that of low LET radiation (e.g., gamma radiation) is 3.05, compared with 3.82 measured during transit primarily due to the shielding variance (Zeitlin, 2013; Hassler et al., 2014). The effective Mars atmospheric shielding is about 21 g/cm^2^, which is much thicker than the spacecraft shielding of the Mars Science Laboratory’s Curiosity Rover during its transportation from Earth to Mars (Zeitlin, 2013; Hassler et al., 2014). For Lunar missions, there is no atmospheric shielding effect. A total estimated mission dose equivalent of ~1.01 Sv was reported for a round trip Mars surface mission consisting of 180 days (each way) and 500 days on the Martian surface for a solar cycle similar to 2011-2012 during the Mars Science Laboratory mission containing the Curiosity rover.

At the cellular level, radiation induces DNA damage, which to some extent is ameliorated by cellular repair machinery. To counteract DNA damage, a network of cellular pathways, defined as the DNA damage response (DDR) network, accommodates moderate DNA damage by detecting and repairing DNA lesions. These mechanisms consist of cell-cycle regulation, DNA repair, and apoptosis. The basic principles of DDR in prokaryotes and eukaryotes are similar, but significant differences exist in the radiosensitivity among different species and the mechanisms that allow access to the lesions by repair enzymes (Arena et al., 2014b). Plants share many features of chromatin organization and DNA repair with fungi and animals (Arena et al., 2014a; Arena et al., 2014b; Donà and Mittelsten Scheid, 2015).

Numerous studies have shown that the effect of ionizing radiation on plants significantly depends upon species, cultivar, development stage, tissue architecture and genome organization, as well as radiation features, e.g., quality, dose, and duration of exposure (reviewed in De Micco et al., 2011). Seeds have been flown on the International Space Station (ISS) and on other LEO venues (Grigoriev et al., 1978; Nevzgodina et al., 1981; Hammond et al., 1996), however, the radiation environment reported in most of these published studies is not comparable to deep-space radiation both in quality and quantity. Furthermore, few studies have examined the impacts of deep-space radiation on seed health, plant growth, or produce quality. Therefore, the mechanisms of early and late responses of plants to radiation exposure, especially space relevant radiation exposure, have not been well characterized. 

In this study, we exposed 10-day-old *Arabidopsis* seedlings to simulated GCR followed by RNA sequencing analysis. *Arabidopsis* is a well-established model system for plant studies. Its genome has been sequenced and most functional genes have been annotated. Therefore, to use *Arabidopsis* is the first step for us to characterize genes and pathways involved in the responses before we can extend investigations to other crop plants which either have not been fully sequenced, or the theoretical genes have not been annotated based on their functions. Highlighted here are the observed transcriptional changes under the selected radiation scenarios.

## Materials and methods

### Plant model organisms and seedling preparation

Wild type *Arabidopsis* seeds (ecotype Columbia-0) were surface sanitized using a standard 70% ethanol method (Massa et al., 2017). Approximately 100 surface sanitized seeds were planted onto 0.5X Murashige and Skoog (MS) phytagel (0.4%) media (with 0.5% sucrose and 1x Gamborg vitamins) in T25 culture flasks and grown in a controlled environment chamber (22°C, 40–45% RH, 150 µmol·m^2^·s^-1^ light, ambient CO_2_ at 400-500 ppm, 16 h/8 h photoperiod) for 8 days. Flasks containing 8 day old plants were shipped overnight at ambient temperature in dark to the NASA Space Radiation Laboratory (NSRL; La Tessa et al., 2016) in Upton, NY. Upon receiving at NSRL, the flasks were wrapped with 4 layers of black tissue paper to prevent light exposure, and stored at room temperature for an additional 24 hrs until the scheduled beam time. The flasks were kept at the same condition during the radiation exposure and the post-irradiation incubation until harvest. Therefore, samples were kept in dark for a total of two days before, during, and after the exposure. Seedlings were 10 days old at time of exposure. All the procedures were carried out at room temperature.

### Radiation scenarios

The *Arabidopsis* seedlings were sequentially exposed to proton, helium, oxygen, silicon, and iron ions to simulate the GCR spectrum (**Table 1**). Exposures were conducted acutely (within minutes for each single ion beam, leading to a total of about 20 mins for all components). Fourteen flasks were subject to either 0 cGy (also referred to as Control; n = 6 flasks), 40 cGy (n = 4), or 80 cGy (n = 4) GCR irradiation. Based on the quality factor of 3.05-3.82, for a 3.5-year mission (1278 days), the total estimated dose equivalent is about 1.47 Sv, or 42.7 cGy. If seeds have to be delivered first to the Mars surface, the actual exposure time could be much longer, possibly close to 6 years, which GCR exposure dose could reach 73.2 cGy. Therefore, we chose 40 cGy and 80 cGy for our investigation.

**Table 1:** Radiation scenario for simulated GCR exposure at the NSRL.

Model Organism	Scenario	Particles	Total Primary Dose Delivered	Total Dose Seedlings Received
10-day *Arabidopsis* Seedlings	Simplified GCR simulation	35% 1000 MeV/n H^1^ + 39% 250 MeV/n H^1^ + 18% 250 MeV/n He^4^ + 6% 350 MeV/n O^16^ + 1% 600 MeV/n Si + 1% 600 MeV/n Fe^56^	40 cGy	40 cGy
			80 cGy	80 cGy

### RNA isolation and RNA sequencing analysis

Three hours after irradiation, immediately after unwrapping the container, seedlings from each flask were harvested and preserved using RNAlater. After 24 hours, preserved samples were transferred to a -80°C freezer. RNA extracts from whole seedlings were isolated from each preserved sample using a homogenizer and the Plant RNeasy Mini Kit (Qiagen), following recommended procedures with on-column DNase treatment. RNA extraction and transcriptomic analysis was performed at Case Western Reserve University's Applied Functional Genomics Core. Libraries were prepared from purified RNA (0.3 µg) using the TruSeq Stranded Total RNA Library Prep with Ribo-Zero Globin (Illumina, 20020613) according to the manufacturer’s protocol. Three runs of 75 bp paired-end sequencing was performed on pooled libraries using the Illumina NextSeq 550 System. Quality control of purified RNA and RNA libraries was carried out using the Fragment Analyzer System (Agilent).

### RNA-seq data processing and identification of differentially expressed genes

RNA sequencing data was processed as described by Overbey et al. (2021). Briefly, raw demultiplexed FASTQ paired-end read files were trimmed of sequencing adapters and low-quality bases using the program TrimGalore (Krueger, 2023). Trimmed reads were then aligned to the *Arabidopsis thaliana* reference genome TAIR10 (release-50) with the STAR aligner (Dobin et al., 2013). Aligned reads were counted and assigned to gene meta-features using the program RSEM (Li and Dewey, 2011). Count files were imported into the R programming environment and analyzed for differentially expressed genes using edgeR (Robinson et al., 2009). Genes with p ≤ 0.01 were considered as genes of interest (GOI), whereas genes with False Discovery Rates (FDR) ≤ 0.1 were identified as differentially expressed genes (DEGs).

For the dose-dependent change in gene expression, logCPM values for genes common to 40 cGy vs. Control and 80 cGy vs. Control comparisons were plotted using ggboxplot function as a part of the ggpubr package available through the Comprehensive R Archive Network (CRAN) (Kassambara, 2023). The significance of dose-dependent change was determined by the Kruskal-Wallis test with p ≤ 0.05 for both individual dose comparisons and the overall comparison. Using the R package clusterProfiler (v4.8.2), DEGs (FDR ≤ 0.1) from 40 and 80 cGy treatments were analyzed for potential enrichment of gene ontology (GO) and KEGG pathway terms at the specified statistical criteria (p value Cutoff = 0.05 and q value Cutoff = 0.3) (Yu et al., 2012; Wu et al., 2021) reported similarly by Tang et al. (2022). Further, the data were also analyzed using methods reported by Neelam et al. (2020) as validation.

Network analysis was accomplished using STRING on both datasets generated from two pipelines (Szklarczyk et al., 2023). The pathway enrichment significance was calculated by STRING using the Benjamini-Hochberg procedure to determine FDR. An FDR less than 0.05 is considered significant. If not specified, the data presented through this article were generated from the pipeline recommended by Overbey et al., 2021.

## Results

### Differentially expressed genes in 10-day-old Arabidopsis seedlings

**Figure 1:**
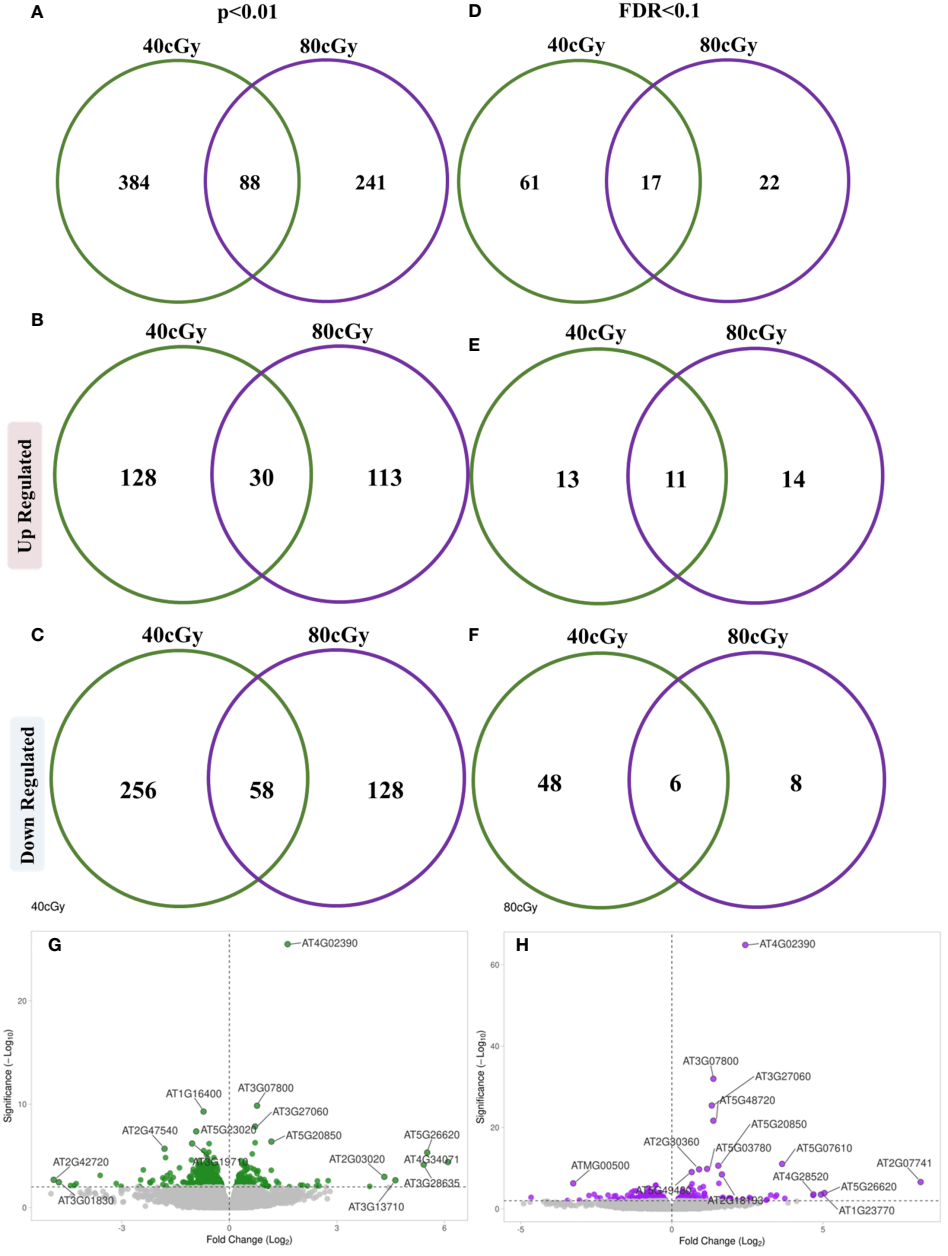
Venn diagrams showing distribution of differentially expressed genes between 40 cGy vs. Control and 80 cGy vs. Control comparisons evaluated using p ≤ 0.01 **(A–C)** and (FDR ≤ 0.1 criterion **(D–F)**. Volcano plots represent differentially expressed genes at a significance cutoff set to p ≤ 0.01 for 40 cGy **(G)** and 80 cGy **(H)** compared to the control (0 cGy).

In this study, 10-day-old *Arabidopsis* seedlings were exposed to simulated GCR irradiation for transcriptomic profile analysis. A total of 472 and 329 genes were found to be differentially regulated (p ≤ 0.01) by 40 and 80 cGy simulated GCR treatment, respectively. While seedlings that received 40 cGy and 80 cGy share a group of 88 GOI, the responses to these two doses of radiation were quite different, showing 384 and 241 genes exclusively in 40 cGy or 80 cGy (**Figure 1A**). Within the up-regulated genes, 128 (47.2%) and 113 (41.7%) genes were exclusive for 40 and 80 cGy treatment, respectively, while 30 (11.1%) genes were shared between both exposures (**Figure 1B**). For down-regulated genes, 40 and 80 cGy irradiation resulted in 256 (57.9%) and 128 (29%) genes with altered expressions exclusively to each treatment with 58 (13.1%) genes shared between both treatments (**Figure 1C**). These results indicated relatively more profound down-regulation of gene expressions in the seedlings exposed to lower dose radiation at 40 cGy.

Using more stringent cutoff criteria with FDR ≤ 0.1, 78 (24 up and 54 down-regulated) and 39 (25 up and 14 down-regulated) DEGs were identified as a result of 40 and 80 cGy exposures, respectively (**Figure 1D**). The distribution of up-regulated DEGs was similar for each treatment showing 13 (out of 24 total up-regulated genes) and 14 (out of 25 total up-regulated genes) genes exclusively for 40 and 80 cGy treatment, respectively, while 11 genes were shared between both exposures (**Figure 1E**). A more profound down-regulated pattern was observed in the 40 cGy group with 48 (out of 54 total down-regulated genes) exclusive DEGs compared to only 8 (out of 14 total down-regulated genes) DEGs in the 80 cGy group. Six down-regulated DEGs were shared between both treatments (**Figure 1F**). In summary, the 40 and 80 cGy treatments revealed expression patterns that are similar in some ways and distinct in others (**Figures 1G**, **H**). All analyses mentioned hereafter were conducted based on the FDR ≤ 0.1 dataset unless indicated specifically.

### Dose-dependent gene expression

**Figure 2:**
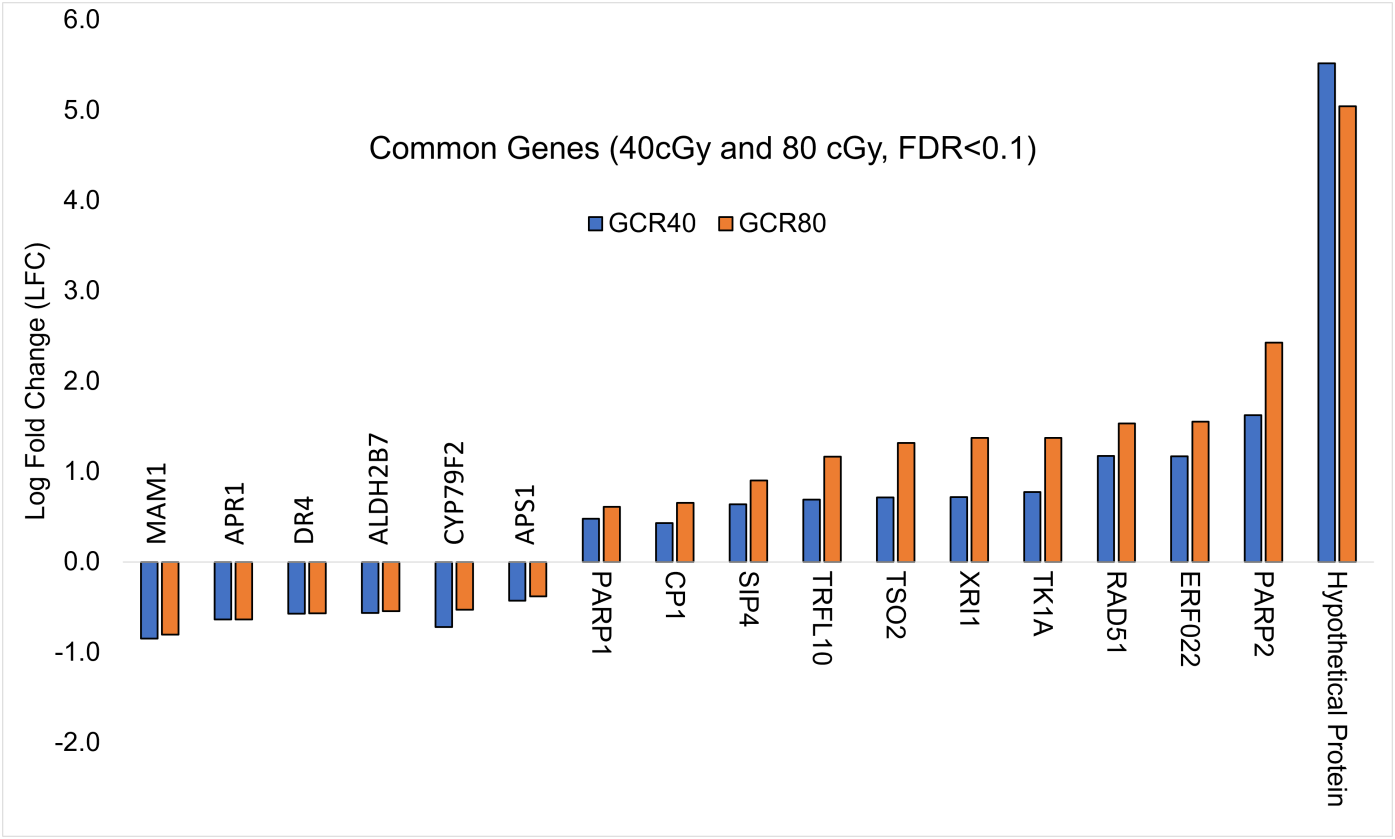
Comparison of log-fold change values (LFC) for DEGs (FDR ≤ 0.1) common to 40 cGy vs. Control and 80 cGy vs. Control comparisons.

**Figure 3:**
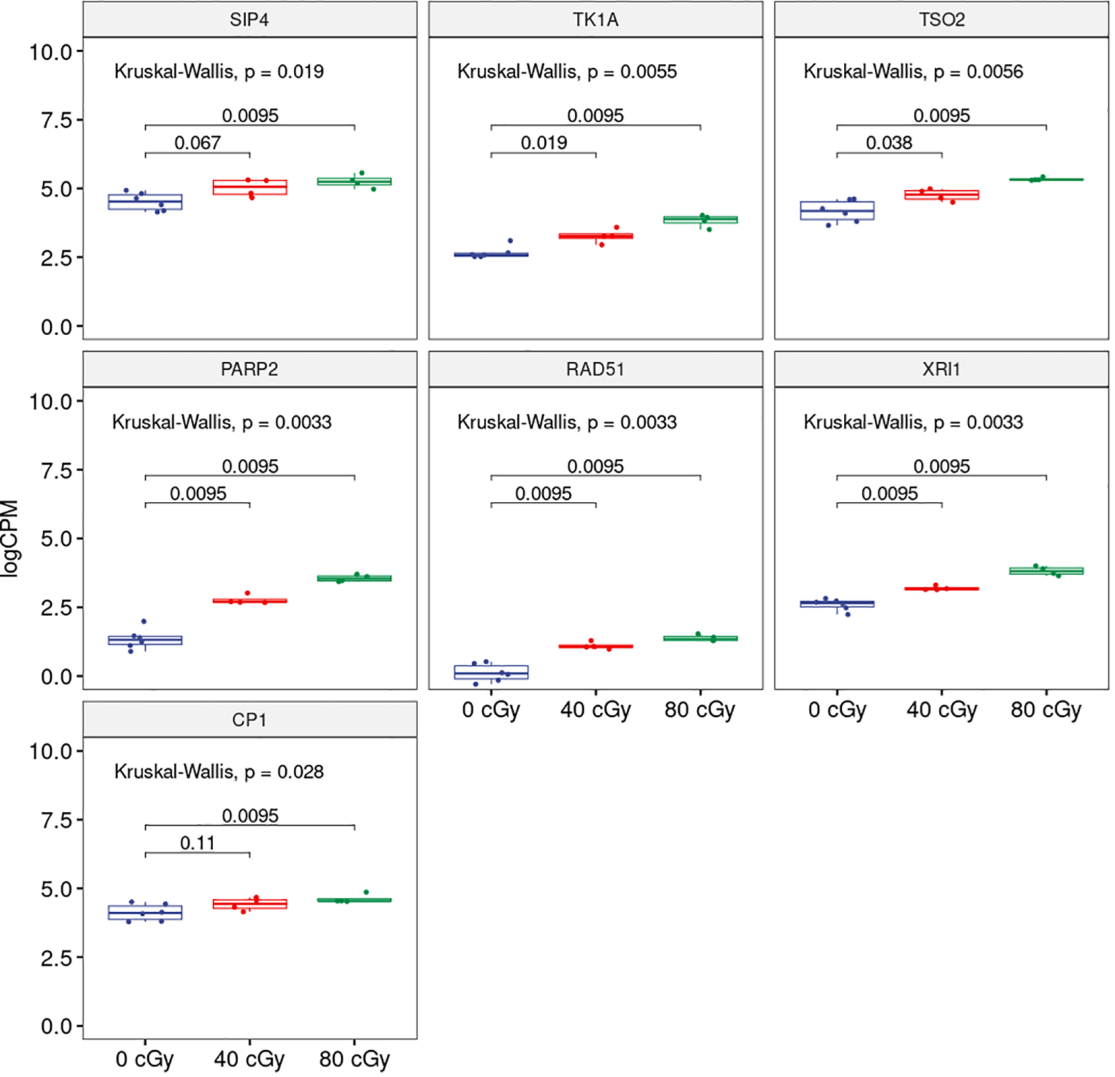
Boxplots showing dose-dependent changes in gene expression among 0, 40 and 80 cGy of simulated GCR exposure. Kruskal-Wallis p-value at the top represents overall statistical significance comparing all three treatments. Horizontal bars spanning 0 and 40 cGy; 0 and 80 cGy compared each of the treatment and the numbers represent statistical significance for each of the comparisons.

To explore further, we analyzed the shared DEGs to determine whether there was any dose-dependent changes in gene expression (**Figure 2**). Among 11 up-regulated DEGs shared by both treatment groups, the expressions of 7 genes showed significant individual dose comparison and dose-dependent response with a much higher expression in the 80 cGy group using Kruskal-Wallis test (p ≤ 0.05). Interestingly, 6 of these genes, including *SIP4* (AT2G30360), *TK1A* (AT3G07800), *TSO2* (AT3G27060), *PARP2* (AT4G02390), *RAD51* (AT5G20850), and *XRI1* (AT5G48720), are involved in DNA damage sensing and DNA repair, while *CP1* (AT5G49480) is involved in cellular defense response (**Figure 3**). In the case of down-regulated genes shared by the 40 and 80 cGy exposures, *CYP79F2* (AT1G16400), *ALDH2B7* (AT1G23800) and *DR4* (AT1G7333) showed statistically significant down-regulation (p ≤ 0.05 by Kruskal-Wallis test) for each dose comparison. However, no dose-dependent down regulation was observed.

### Unique responses in 40 cGy and 80 cGy treatments

To further elucidate effect(s) of each of the simulated radiation dosage and identify any unique responses to the simulated GCR exposure, we looked at the genes whose expression was exclusively modulated due to 40 or 80 cGy irradiation. Among the 61 genes exclusively regulated by 40 cGy exposure, 12 were up-regulated including *ACS11* (AT4G08040) and *BG1* (At5G12050) (**Supplemental Table 2**). Among 48 down-regulated genes, 6 genes, namely *FMO GS-OX1* (AT1G65860), *BCAT4* (AT3G19710), *DJ1F* (AT3G54600), *BAT5* (AT4G12030), *IMD1* (AT5G14200), and *MAM3* (AT5G23020) are part of the glucosinolate biosynthetic pathway which was found to be enriched. *BGLU5* (AT1G60260) and *CAR7* (AT1G70810) were involved in glucosinolate metabolic processes. In addition, 12 genes (marked with an * in the supplemental table) had been reported to be involved in defense and cellular responses to environmental stimuli, such as responses to wounding, bacteria, heat, drought, light stimuli, hypoxia, oxidative stress, and ethylene. Some of these genes may also belong to glutathione and methionine pathways (**Supplemental Table 3**).

Exposure to 80 cGy had 15 up-regulated exclusive genes. Notably, among these genes, *SYN2* (AT5G40840), *TIL1* (AT1G08260), AT1G49980 (a DNA/RNA polymerase superfamily protein), *BRCA1* (AT4G21070), *PARP1* (AT2G31320), and *RAD17* (AT5G66130) had either a known or putative function involved in DNA damage repair. Other genes up-regulated due to 80 cGy exposure included AT5G48020 (2-oxoglutarate (2OG) and Fe (II)-dependent oxygenase), AT5G60250 (zinc finger (C3HC4-type RING finger) family protein), and AT2G18193 (P-loop containing nucleoside triphosphate hydrolases). Genes exclusively down-regulated due to 80 cGy exposure included *MSRB5* (AT4G04830; Methionine sulfoxide reductase B5), *MOS9* (AT1G12530; Modifier of SNC1 9) and *NP2* (AT1G54960; NPK1-related protein kinase 1). Clearly, exposure to 40 and 80 cGy exhibited some unique responses from the 10-day-old seedlings (**Supplemental Tables 4**, **5**).

### Pathway and network analysis

Analysis of the up regulated genes from 40 cGy exposure showed GO functional enrichment of processes associated with DNA repair, cellular response to DNA damage stimulus and response to ionizing radiation, whereas the down-regulated genes showed an enrichment of processes related to sulfur compound biosynthetic processes and S-glycoside metabolic processes (**Table 2**). Similar to 40 cGy exposure, genes up-regulated by 80 cGy exposure showed GO functional enrichment of processes associated with DNA repair, response to ionizing radiation, condensed nuclear chromosomes, and the down-regulated genes also showed an enrichment of functional processes related to S-glycoside metabolic processes, and glucosinolate metabolic processes (**Table 3**).

**Table 2:** GO enrichment for DEGs (FDR ≤ 0.1) from 40 cGy vs. Control comparison.

	Description	Adjusted p-value	Gene ID
**Up-regulated genes**	DNA repair	3.51E-06	AtTK1a, ATTSO2, APP, ATRAD51, XRI
	cellular response to DNA damage stimulus	3.51E-06	AtTK1a, ATTSO2, APP, ATRAD51, XRI
	response to ionizing radiation	9.00E-04	ATRAD51, XRI
	meiotic nuclear division	1.33E-02	ATRAD51, XRI
	DNA replication	1.90E-02	ATTSO2, ATRAD51
	nuclear division	2.04E-02	ATRAD51, XRI
	organelle fission	2.04E-02	ATRAD51, XRI
**Down-regulated genes**	sulfur compound biosynthetic process	1.47E-07	CYP79F2, BCAT4, APS1, GGP1, GSM1, IMS2
	secondary metabolite biosynthetic process	1.47E-07	CYP79F2, ATGRP9, BCAT4, GGP1, GSM1, IMS2
	S-glycoside metabolic process	1.47E-07	CYP79F2, NA, BCAT4, GGP1, GSM1, IMS2
	glycosinolate metabolic process	1.47E-07	CYP79F2, NA, BCAT4, GGP1, GSM1, IMS2
	glucosinolate metabolic process	1.47E-07	CYP79F2, NA, BCAT4, GGP1, GSM1, IMS2
	glycosyl compound metabolic process	4.49E-07	CYP79F2, NA, BCAT4, GGP1, GSM1, IMS2
	S-glycoside biosynthetic process	4.49E-07	CYP79F2, BCAT4, GSM1, IMS2
	glucosinolate biosynthetic process	4.49E-07	CYP79F2, BCAT4, GSM1, IMS2
	glycosyl compound biosynthetic process	9.25E-07	CYP79F2, BCAT4, GSM1, IMS2
	branched-chain amino acid metabolic process	4.73E-05	BCAT4, GSM1, IMS2
	leucine biosynthetic process	3.92E-04	GSM1, IMS2
	leucine metabolic process	8.79E-04	GSM1, IMS2
	amino acid transmembrane transport	1.56E-03	SIAR1, GDU1
	export across plasma membrane	1.56E-03	SIAR1, GDU1
	regulation of small molecule metabolic process	1.56E-03	NA, DJ-1f
	acyltransferase activity, acyl groups converted into alkyl on transfer	1.56E-03	GSM1, IMS2

**Table 3:** GO enrichment for DEGs (FDR ≤ 0.1) from 80 cGy vs. Control comparison.

	Description	Adjusted p-value	Gene ID
**Up-regulated genes**	DNA repair	2.83E-08	PARP1, AtTK1a, AtTSO2, APP, AtBRCA1, AtRAD51, AtRAD21.1, XRI
	cellular response to DNA damage stimulus	3.23E-08	PARP1, AtTK1a, ATTSO2, APP, ATBRCA1, ATRAD51, AtRAD21.1, XRI
	response to ionizing radiation	7.81E-05	ATBRCA1, ATRAD51, XRI
	meiotic nuclear division	3.15E-03	ATRAD51, AtRAD21.1, XRI
	nuclear division	1.11E-02	ATRAD51, AtRAD21.1, XRI
**Down-regulated genes**	S-glycoside metabolic process	3.65E-02	CYP79F2, ATMSRB5
	glycosinolate metabolic process	3.65E-02	CYP79F2, ATMSRB5
	glucosinolate metabolic process	3.65E-02	CYP79F2, ATMSRB5
	glycosyl compound metabolic process	4.27E-02	CYP79F2, ATMSRB5

**Figure 4:**
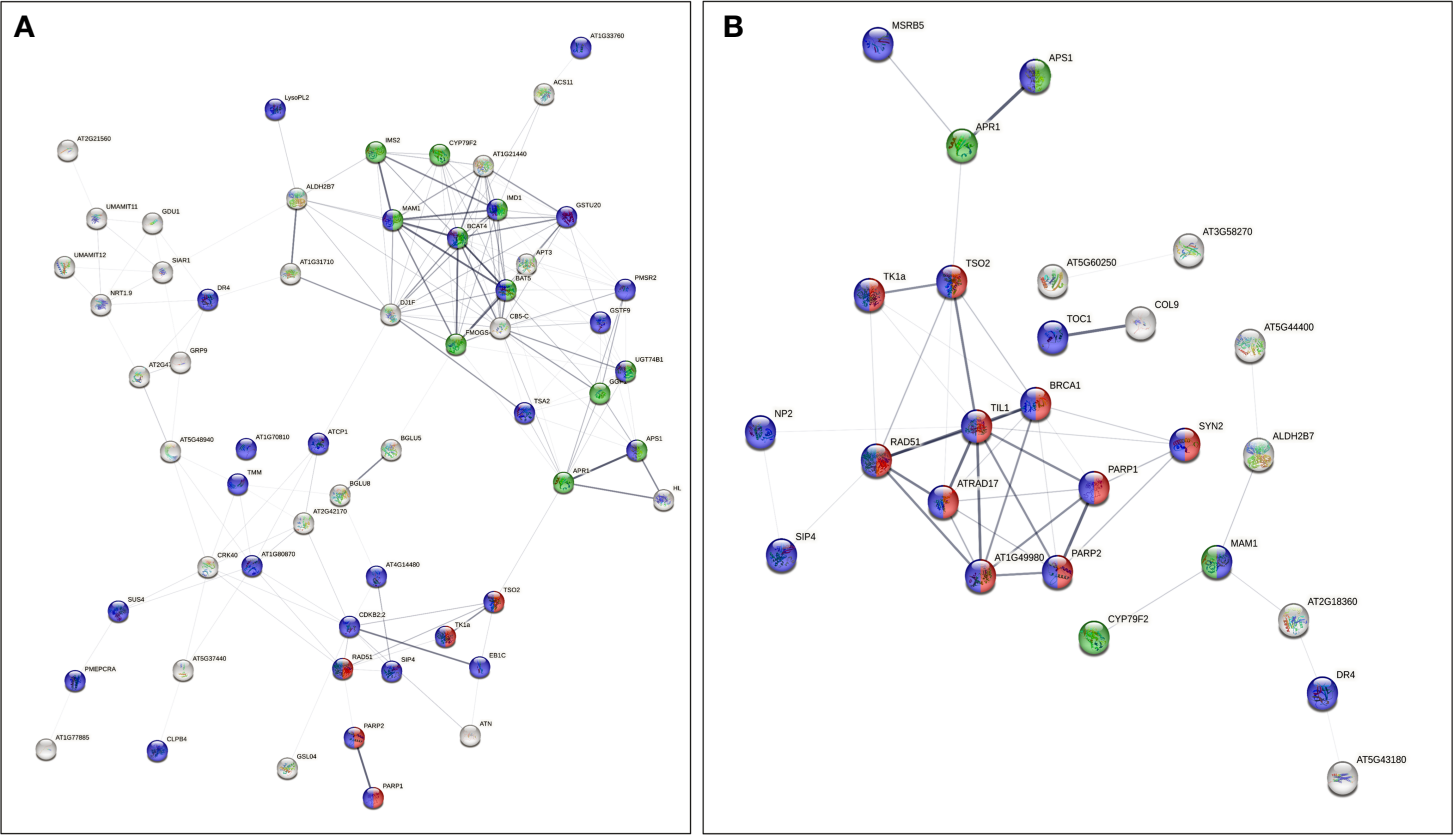
Gene interaction networks from genes of interest (FDR ≤ 0.1) identified by STRING. Color coded nodes show DNA repair (red), glucosinolate biosynthesis pathways (green), and genes involved in responses to stimuli (purple) in plants exposed to **(A)** 40 cGy and **(B)** 80 cGy. Network edges indicate co-occurrence, co-expression, and evidence from experiments and databases. Network edge width indicates the confidence or strength level from 0.17 to 2.56 (40 cGy) and 0.28 to 2.85 (80 cGy).

The analysis also showed a statistically significant enrichment of KEGG pathways associated with pyrimidine and nucleotide metabolism pathways from the up-regulated genes and an enrichment of glucosinolate biosynthesis, 2-oxocarboxylic acid metabolism pathways due to down-regulated genes from 40 cGy exposure. Genes up-regulated due to 80 cGy also showed an enrichment of KEGG pathways associated with base excision repair, homologous recombination, pyrimidine metabolism. Down-regulated genes on the other hand did not reveal any KEGG pathway enrichment within specified statistical criteria (**Table 4**). Network analysis using STRING showed consistent results with clustered gene interaction groups particularly in DNA repair, glucosinolate biosynthesis, and sulfate assimilation pathways (**Figures 4A, B**).

**Table 4:** KEGG enrichment of DEGs (FDR ≤ 0.1).

		ID	Description	Adjusted p-value	Gene ID
**40 cGy vs. Control**	Up-regulated	ath00240	Pyrimidine metabolism	4.90E-03	AtTK1a, ATTSO2
		ath01232	Nucleotide metabolism	4.90E-03	AtTK1a, ATTSO2
	Down-regulated	ath00966	Glucosinolate biosynthesis	7.00E-07	CYP79F2, BCAT4, GSM1, IMS2
		ath01210	2-Oxocarboxylic acid metabolism	2.94E-05	CYP79F2, BCAT4, GSM1, IMS2
**80 cGy vs. Control**	Up-regulated	ath03410	Base excision repair	1.38E-02	PARP1/APP
		ath00240	Pyrimidine metabolism	1.38E-02	AtTK1a/ATTSO2
		ath03440	Homologous recombination	1.38E-02	ATBRCA1/ATRAD51
		ath01232	Nucleotide metabolism	1.38E-02	AtTK1a/ATTSO2

## Discussion

Previously, it was found that GCR impact hydrated *Arabidopsis* seeds in a dose dependent and quality dependent manner (Zhang et al., 2022). To complement that study and to broaden the understanding of plant response to radiation exposure, 10-day-old *Arabidopsis* seedlings were exposed to simulated GCRs. Seedlings were exposed to either 0 cGy, 40 cGy, or 80 cGy acute doses of GCR, kept for 3 hours, then preserved for RNA extraction. The reason for choosing 10 days old seedlings was mainly because 1) Seedlings are in the rapid growing stage with 2 rosette leaves emerged, which are potentially more sensitive to radiation. Therefore, we could see more changes to identify genes and pathways; 2) Considering the logistics of doing a simulated radiation exposure experiment, the seedling size at this age is very manageable; and 3) 10 days old seedlings were used for many spaceflight investigations, which make the result comparison possible for further analysis. The 40 cGy and 80 cGy treatments were chosen because they approximate possible exposures for a long-duration deep space mission, e.g., a 3-year Mars expedition. Providing uniform light to treatment and control samples was not possible within the radiation facility, so plants were kept in the dark for a total of 2 days prior, during, and after irradiation until their harvest and fixation. These methods eliminated light variance between experimental treatments, which potentially affect DNA repair efficiency because light regimes have been shown to dictate the specific activation and efficiency of certain DNA repair pathways, such as DNA recombination or photo repair in various plants (Li et al., 2002; Boyko et al., 2005; Bray and West, 2005; Chang et al., 2008; Manova and Gruszka, 2015; Nisa et al., 2019).

Our primary goal was to elucidate the DNA damage sensing and repair machinery in *Arabidopsis* involved in the responses to GCR irradiation. We chose to use acute simulated GCR exposure (80 cGy in 20 mins) to generate valuable baseline data to enable us to conduct cross species comparison and to better interpret data obtained from irradiation experiments at lower dose-rate (80 cGy in 4 hrs) in our other studies. GCR is always present at an extremely low dose rate in deep space. However, it is impossible to simulate GCR exposure on Earth using both comparable particle types and dose rate. Thus, the data presented here using relevant GCR particles is still valuable for deep space radiation impact assessment. We chose the post-irradiation time-point (3 hrs) based on extensive data (e.g., gamma H_2_AX and gene expression patterns) collected from other organisms such as mammalian cells and small animal models as well as limited data from studies using *Arabidopsis* plants in response to radiation exposure (Penninckx et al., 2021). Interestingly, our data revealed a dose-dependent response especially in the up-regulated genes, indicating that these changes are specific to radiation exposure. Further enrichment analysis revealed some expected changes, such as DNA repair mechanisms, but also revealed some metabolic changes that may have implications for space agriculture.

### DNA repair pathway in response to simulated GCR exposure

With few exceptions, plants share most of the common DNA repair components first described in other eukaryotic systems, such as yeast and mammals (Britt, 2002; Arena et al., 2014b; Donà and Mittelsten Scheid, 2015). Photoreactivation is one of the primary DNA repair mechanisms needed by plants on a daily basis to repair UV-induced DNA damage in real-time while the plants are exposing to solar light. The two classical forms of excision repair, base excision repair (BER) and nucleotide exchange repair (NER), as part of “dark repair”, are also available for the plant to deal with various types of DNA lesions (Rastogi et al., 2010). DNA mismatch repair (MMR) is responsible for the removal of incorrectly paired nucleotides and the UV-induced photolesions from the genome of higher plants (Culligan and Hays, 2000; Lario et al., 2011). The main DNA double-strand break repair pathways – homologous recombination (HR) and nonhomologous end joining (NHEJ) have been shown to be essential in plants for the preservation of their genetic stability (Puchta et al., 1996; Waterworth et al., 2011). Some of the repair mechanisms such as photoreactivation are highly specialized for a particular type of damage, generated from a specific source, however, others like excision and recombination pathways may deal with a variety of lesions (Ries et al., 2000).

Our data indicated that, at 3 hrs post irradiation, DNA repair was one of the most prominent responses based on ontology term enrichment, and all genes relating to it were up-regulated. The response was dose-dependent, and the differentially expressed genes belong to diverse aspects of the DNA repair response, including DNA synthesis, double-strand break (DSB) repair, and BER. Among the most thoroughly studied DNA repair genes in *Arabidopsis*, *RAD51*, *PARP1, and PARP2* were identified in our study. In *Arabidopsis*, *RAD51* is a single-copy gene, homologous to yeast Rad51, and transcriptionally activated after gamma-irradiation (Doutriaux et al., 1998). The *PARP* genes in *Arabidopsis* are directly homologous to *PARP* genes in humans and serve a very similar function (Taipakova et al., 2020). Specifically, PARP proteins are activated by and bind to DNA strand breaks. PARP1 (or PARP2) then ribosylates the break termini, which recruits further DNA repair machinery. PARP has previously been reported as up-regulated in response to ionizing radiation in *Arabidopsis* (Doucet-Chabeaud et al., 2001; Ryu et al., 2018) and other plant species (Arena et al., 2014a). PARPs are involved in DSB repair process, especially the NHEJ pathway (Charbonnel et al., 2011; Jia et al., 2013). The experimental conditions used in these studies (e.g., extremely high radiation doses at 10s to 100s Gy) were dramatically different from the conditions we used for this study, which were only at cGy level. Surprisingly, our data showed dose-dependent increase in the expression of *PARP2/APP* and *RAD51* indicating at lower dose range DNA repair pathways is activated in plants. In mammalian cells, the expression of these proteins and their proper functions are closely associated with ATM (Huber et al., 2004; Aguilar-Quesada et al., 2007). Thus, ATM may also play a critical role in Arabidopsis's response to simulated GCR. However, we did not observe changes in ATR’s expression as well as putative ATR responsive genes, indicating ATR may not be involved in plant’s responses to radiation exposure at the radiation levels and timepoints we tested. Moreover, because the DNA damage sensing and repair pathways have not fully illustrated in *Arabidopsis*, we cannot rule out ATR’s involvement unless it is confirmed by ATR phosphorylation analysis.

Additionally, network analysis using STRING on DEGs in 80 cGy response identified another five DEGs, namely ATRAD17, BRCA1, TSO2, TIL1, and AT1G49980, which were potentially regulated by ATM in plants. This assumption is mainly based on their putative homologs found interacting in other organisms (**Figure 4**). ATM has been proposed to be one of the key upstream regulators in DNA damage response in *Arabidopsis* to high dose irradiation (Culligan et al., 2006). Our data indicate that ATM may also play a key role in the responses to lower dose charged particle exposure. BRCA1 is required for the efficient repair of DSB by homologous recombination in somatic cells of *Arabidopsis* (Trapp et al., 2011). BRCA1 colocalizes with RAD51 at sites of DNA damage/replication and activates RAD51-mediated homologous recombination repair of DSB. AT1G49980 is homologous to DINB gene which was found in yeast first (*DinB*), and later in human and mouse. It is a member of error-prone bypass of certain DNA lesions by the human DNA polymerase kappa (Ohashi et al., 2000). These up-regulated DEGs indicated that both the error-prone DNA repair mechanism and homologous recombination repair were involved in the responses to simulated GCR exposure.

At the early response stage analyzed in our study, biological organisms primarily respond to radiation exposure induced direct damages, such as DNA strand breaks and other types of DNA damages, as well as free radicals generated directly by ionizing. These acute responses are dominantly involved with up-regulation of DNA sensing and repair genes, which are radiation dose responsive. In contrast, down-regulated gene expression patterns are less dose responsive, indicating more pathways, such as adaptive, survival/cell death, and metabolism, other than DNA damage responsive pathways, may be involved in modulating the responses.

### Glucosinolate Pathways

**Figure 5:**
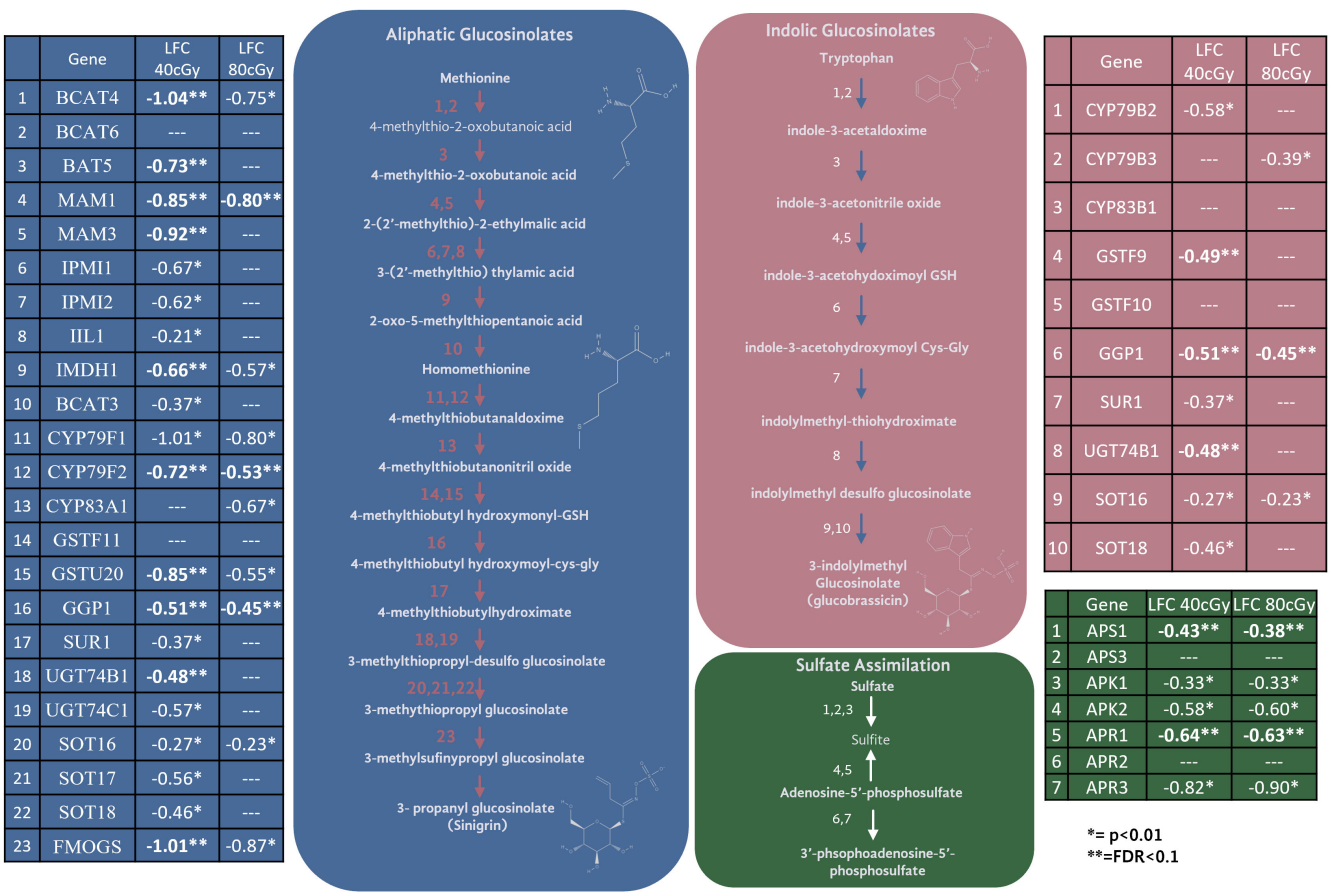
Genes and their expression changes in glucosinolate biosynthesis and sulfate assimilation pathways.

The second most prominent trend in these data is a clear and pronounced downregulation of the glucosinolate biosynthetic pathway at 40 cGy, and in particular the aliphatic glucosinolates derived from methionine (**Figure 5**). The expression of approximately half of the genes required for glucosinolate production from methionine show down-regulation with FDR ≤ 0.1 (pathway enrichment FDR=5.5e-10) at 40 cGy. Furthermore, with a relaxed statistical cutoff of p<0.01, the expression of nearly every enzyme in the pathway is down-regulated at 40 cGy (pathway enrichment FDR=4.10e-15). This includes the sulfate assimilation genes APR, APS, and APK, which are considered part of the glucosinolate biosynthetic network (Yatusevich et al., 2010). In contrast, 80 cGy exposure showed enrichment of the glucosinolate pathway only when analyzing the gene dataset with p<0.01 cutoff (pathway enrichment FDR=7.62e-8), but not from the more stringent dataset using FDR ≤ 0.1 cutoff.

Glucosinolates are a class of sulfur-containing secondary metabolites which play an important role in plant defense. They are produced in sixteen plant families representing nearly 5,000 plant species (Chhajed et al., 2020). Glucosinolate metabolism responds to a multitude of environmental factors such as salt, wounding, herbivory, and UV exposure (Chhajed et al., 2020; Chowdhury, 2022; Martínez-Ballesta et al., 2013; Wang et al., 2011). As glucosinolates serve as defense molecules, their production is often up-regulated in response to abiotic stress. However, the dynamics of how environmental stressors impact the metabolism of specific glucosinolate pathways is not fully resolved, and severe stress conditions can also cause decreased glucosinolate content (Martínez-Ballesta et al., 2013).

The limited time-course studies available reveal the upregulation of glucosinolate content and biosynthetic pathway transcripts within one hour of exposure to prolonged UV-B exposure, where downregulation responses occur 3 hours after stimulus (Wang et al., 2011). The downregulation may be related to the modulation of glucosinolate biosynthesis after the early initial upregulation of the pathway in response to the sudden influx of reactive oxygen species (ROS) in plant tissues. In general, the DNA repair process is radiation quality, quantity, and fluence dependent, and peaked in damaged cells within 4 hours after radiation exposure. According to immunofluorescence analysis, in mammalian cells after radiation exposure, gamma-H2AX (gH2AX) foci appear in DNA damaged cell nuclei within 30 min after exposure. The gH2AX foci then colocalize with other DNA repair machinery proteins, such as P53BP1, RAD50, RAD51, BRCA1, RPA and many others, and such colocalizations typically reach peak within 4 hours, indicating ongoing repair activities (Paull et al., 2000; Penninckx et al., 2021). At 4 hr to 8 hr post exposure, the foci number and intensity start to decrease, indicating the completion of most of the repairs. Our study only used one timepoint, which is not sufficient to examine the possibility of crosstalk between glucosinolate and DNA repair pathways, however it presents an intriguing direction for future work. The glucosinolate pathway may modulate or be modulated with DNA damage sensing and repair, and its kinetics is likely associated with the DNA repair mechanism in response to the ROS caused by radiation exposure. The modulation of glucosinolate content and pathways can also be triggered by light/dark light regime changes. However, light exposure was tightly controlled in this study. All the samples were kept in dark before, during, and after irradiation, so changes in glucosinolate-related gene expression is unlikely related to light exposure. Furthermore, under different light and light cycle conditions, the involvement of glucosinolate pathways and their responses could be greatly different.

Two of the key genes down-regulated in the glucosinolate pathway were glutathione S-transferases, which may suggest the downregulation of glucosinolates as a means to preserve glutathione. Exposure to ionizing radiation increases the oxidative stress experienced by the plant. The production of many glucosinolates requires glutathione, as glutathione is conjugated to intermediate glucosinolate compounds during synthesis. Glutathione serves as a powerful antioxidant under radiation conditions, and the downregulation of the glucosinolate biosynthetic pathway may indicate a prioritization to use glutathione in its role as an antioxidant under these conditions. However, there is no clear evidence of transcriptomic activation of glutathione biosynthetic pathways or other oxidative stress responses. While glutathione is a known player in radiation response, much more biochemical data would be needed to confirm its relationship to glucosinolate production and balancing redox stress. Of the two primary glucosinolate production pathways (aliphatic using methionine as a precursor, and indolic using tryptophan as a precursor), glucosinolate production from methionine was especially enriched among down-regulated genes. Cellular methionine can serve to protect cells against radiation damage, and radiation depletes cellular methionine (Luo and Levine, 2009). The trends observed in these data may suggest cellular effort to preserve methionine after radiological depletion. Among its many critical roles, methionine is central to cellular “one-carbon metabolism” which, among other things, contributes to epigenetic mechanisms through DNA methylation (Miousse et al., 2017). Epigenetic changes have indeed been observed in plants exposed to spaceflight (Xu et al., 2018), and therefore, during long-duration deep space missions within higher space radiation environments, possible disruption in available methionine may have greater impact on epigenetic regulations. The relationship between methionine, radioprotection, one-carbon metabolism, and epigenetics is an intriguing direction of investigation for the future of spaceflight.

While the downregulation of the glucosinolate pathway is noteworthy in the number of genes down-regulated, the changes were not drastic in magnitude (most changes were between LFC of -0.5 and -1). Glucosinolate production is controlled by a handful of transcription factors, but the most impactful in the pathways are MYB28 and MYB29. While these genes did not meet the cutoff for FDR of ≤ 0.1, MYB29 did show downregulation under a relaxed p-value in both treatments (40 cGy LFC= -1.1, p=0.003. 80 cGy LFC=-0.91, p=0.008). Interestingly, the trend appears stronger in the 40 cGy treatment than the 80 cGy treatment. It is unclear why the lower dose of radiation produced a more pronounced effect in the downregulation of glucosinolate biosynthetic pathways. It is possible that the 80 cGy treatment saw a similar change in glucosinolate downregulation, but the change was transient and happened more quickly at the higher dose. Time course analysis would be necessary to determine how dose impacts the temporal dynamics of changes in gene expression. The significance of glucosinolate biosynthesis and its crosstalk with the DNA repair mechanisms need to be further elucidated.

Regardless of the regulatory mechanism, the downregulation of glucosinolates holds important implications for plants growing in the radiation environment of space. Firstly, glucosinolates offer plants protection from infection by bacteria and fungi through enzymatic conversion from glucosinolates to isothiocyanates. Importantly, plants demonstrate an increased susceptibility to infection in spaceflight (Leach et al., 2001). A decrease in production of glucosinolates in response to radiation presents an intriguing mechanism for a plant’s lowered capacity to ward off pathogenic infection under spaceflight conditions. Moreover, the same isothyocyanates that convey pathogen resistance to plants are also the compounds that imbue the desirable spicy flavor to radishes and many other cruciferous vegetables. Therefore, a downregulation in glucosinolate production not only impacts a plant’s survivability but may also impact its flavor profile. Of course, these treatments were delivered acutely, and these impacts may or may not be observed at lower radiation dose rates.

### Expanded network analysis to explore other potential radiation responsive pathways

To explore as much as possible *Arabidopsis* pathways involved in responses to simulated GCR exposure, we expanded the pathway analysis using genes of interest (p<0.01) datasets generated from both bioinformatics pipelines. Using STRING GO enrichment analysis, we found 86 and 37 GO processes enriched for 40 cGy and 80 cGy treatments, respectively from both datasets. Among these functional enrichments, 24 GO enrichment groups were shared by the two dose treatments (**Table 5A**). There are 13 GO enrichment groups unique for the 80 cGy treatment (**Table 5B**), mainly in DNA repair and metabolic processes and cell cycle regulations. For the 40 cGy treatment, a total of 62 unique GO processes were identified, including the response to hormones and defense responses. In the following sections, we discuss more on some of these identified unique processes other than DNA repair and glucosinolate related pathways.

**Table 5A:** GO processes shared between the 40 cGy and 80 cGy treatments.

#Category	Term ID	Term Description
GO Process	GO:0044550	Secondary metabolite biosynthetic process
GO Process	GO:0019748	Secondary metabolic process
GO Process	GO:0019760	Glucosinolate metabolic process
GO Process	GO:0019761	Glucosinolate biosynthetic process
GO Process	GO:1901657	Glycosyl compound metabolic process
GO Process	GO:0000103	Sulfate assimilation
GO Process	GO:0044272	Sulfur compound biosynthetic process
GO Process	GO:0006790	Sulfur compound metabolic process
GO Process	GO:0000097	Sulfur amino acid biosynthetic process
GO Process	GO:1901605	Alpha-amino acid metabolic process
GO Process	GO:1901607	Alpha-amino acid biosynthetic process
GO Process	GO:0006534	Cysteine metabolic process
GO Process	GO:0006520	Cellular amino acid metabolic process
GO Process	GO:0050896	Response to stimulus
GO Process	GO:0042221	Response to chemical
GO Process	GO:0051716	Cellular response to stimulus
GO Process	GO:0033554	Cellular response to stress
GO Process	GO:0006950	Response to stress
GO Process	GO:0009719	Response to endogenous stimulus
GO Process	GO:0009628	Response to abiotic stimulus
GO Process	GO:0010033	Response to organic substance
GO Process	GO:1901700	Response to oxygen-containing compound
GO Process	GO:0065007	Biological Regulation
GO Process	GO:0065009	Regulation of molecular function

**Table 5B:** Unique GO processed enriched by 80 cGy treatment.

#Category	Term ID	Term description
GO Process	GO:0007049	Cell cycle
GO Process	GO:0000278	Mitotic cell cycle
GO Process	GO:0006281	DNA repair
GO Process	GO:0006302	Double-strand break repair
GO Process	GO:0006974	Cellular response to DNA damage stimulus
GO Process	GO:0006259	DNA metabolic process
GO Process	GO:0009700	Indole phytoalexin biosynthetic process
GO Process	GO:0050794	Regulation of cellular process
GO Process	GO:0010564	Regulation of cell cycle process
GO Process	GO:0080090	Regulation of primary metabolic process
GO Process	GO:0051171	Regulation of nitrogen compound metabolic process
GO Process	GO:0031323	Regulation of cellular metabolic process
GO Process	GO:0050789	Regulation of biological process

### Cell cycle regulation interacting with DNA repair pathways

**Figure 6:**
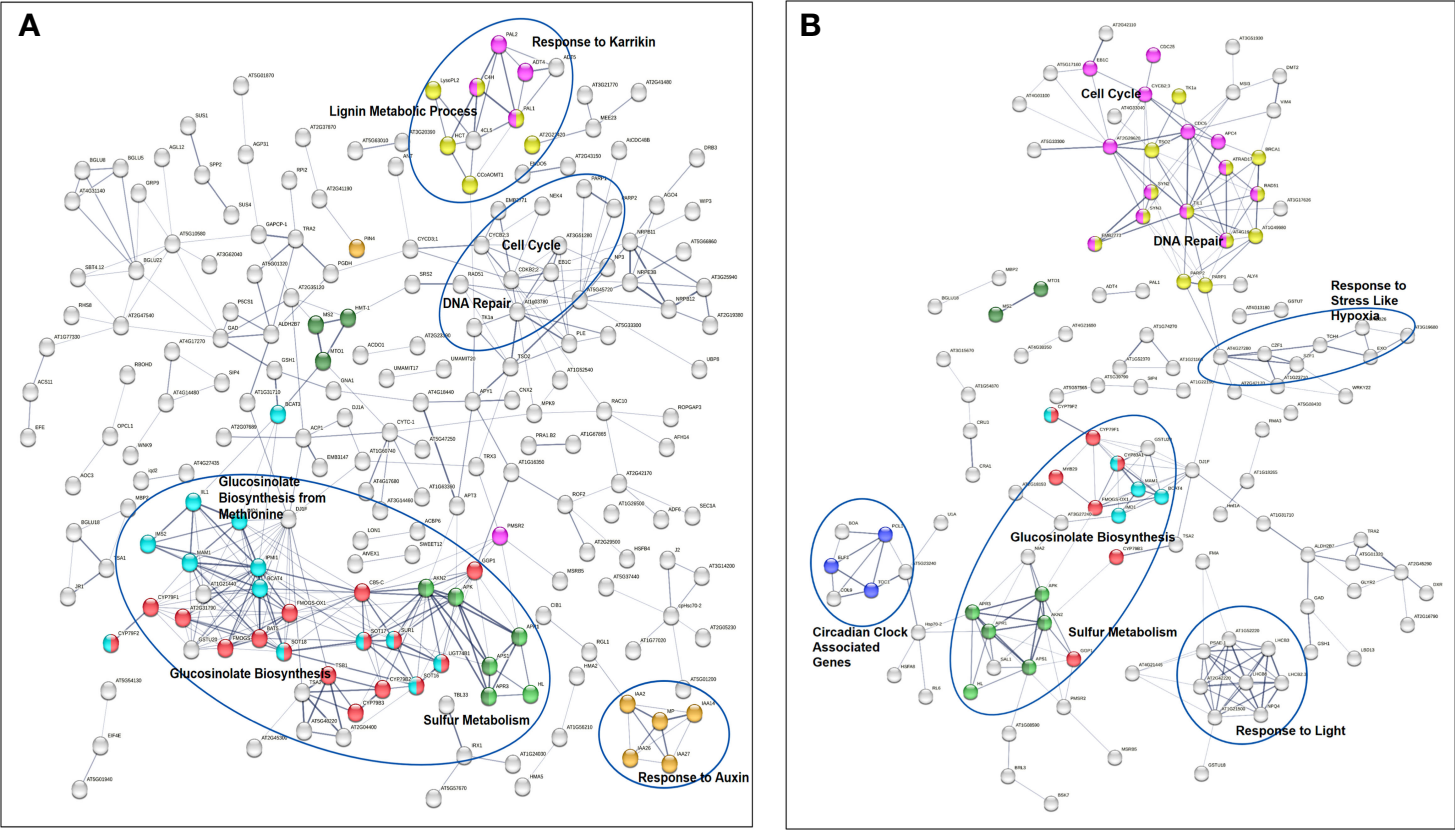
Gene interaction networks from genes of interest (p ≤ 0.01) identified by STRING. Network edges indicate co-occurrence, co-expression, and evidence from experiments and databases. Network edge width indicates the confidence and strength level higher than 0.4. Color coded nodes show glucosinolate biosynthesis and cellular response to sulfur starvation (red), glucosinolate biosynthesis from methionine (light blue), sulfate assimilate (green), and sulfur metabolism and methionine metabolic process (dark green) in both **(A)** 40 cGy and **(B)** 80 cGy treatments. Dose dependent responses include more hormone related responses in the 40 cGy treatment (yellow, pink, and orange). In contrast, the 80 cGy treatment has more enriched gene clusters in DNA repair (pink), cell cycle (yellow), and circadian clock (purple) pathways. Genes related to DNA repair and cell cycle are highlighted in a circle in the 40 cGy treatment, and genes responding to hypoxia and light are highlighted in circles in the 80 cGy treatment.

Cell cycle progression is tightly regulated during DNA repair. Compared to 40 cGy, 80 cGy exposure caused more cell cycle checkpoint regulations (**Figure 6**). Many DNA repair genes are involved in cell cycle progression, such as *RAD51* and *RAD17*. The expanded network analysis identified *CDC6*, *TIL1*, *At4G19130, EMB2773*, *SYN2*, *SYN3*, *CYCB2;3*, *AT2G28620*, *EB1C*, and *CDC25*, to be closely related to DNA repair responses. *CDC6*, *At4G19130*, *TIL1*, *SYN2*, and *SYN3* are potentially directly associated with *RAD5*1 and *RAD17* in the crosstalk between DNA repair and cell cycle progression control. *CDC6* is an essential regulator of initiating DNA replication based on the functions of its homologous counterpart in other eukaryotic cells, therefore it may play important roles in the activation and maintenance of the S and mitosis checkpoint in *Arabidopsis*. *At4G19130* encodes replication protein A (RPA) 70 kDa component required by both DNA repair and cell cycle control through its activity in DNA recombination and replication. We recommend to officially name *At4G19130 RPA* based on the evidence of its responsiveness to radiation exposure. *TIL1* encodes the catalytic subunit of DNA pol ϵ of *Arabidopsis* (Jenik et al., 2005). Interestingly our data didn’t indicate any changes in the expression of *TED* which encodes *Arabidopsis* DNA polymerase θ. *SYN2* and *SYN3* are homologs to the *RAD21* family in many other organisms. RAD21 has been shown to directly interact with RAD17 and other DNA repair machinery proteins as well as being involved in in mitosis processes.

In addition to these key players in the crosstalk between DNA repair and cell cycle progression control, simulated GCR exposure also resulted in the upregulation of *CDC25*, *CYCB2;3*, *At2G28620*, *At5G33300*, *EMB2773*, and *EB1C*. CDC25 and CYCB2;3 (encoding cyclin B2) may control entry into and progression through various phases of the cell cycle, especially M2/mitosis and mitosis/S (“Synthesis”) phases, similar to their homologs in other organisms. In human, the B-type cyclins, B1 and B2, are part of essential components of the cell cycle regulatory machinery. Proteins encoded by *AT2G28620, At5G33300*, and *EB1C* are associated with microtubule organization, cytokinesis, and the mechanisms to stabilize the mitotic spindle, therefore being involved in mitosis. At2G28620 is also called RSW7, homologous to the KIF family, especially KIF7. AT5G33300 encodes chromosome-associated kinesin-like protein, encoding an ATP dependent microtubule-based motor protein. EMB2773 is involved in centromere complex assembly and mitotic sister chromatid cohesion.

### Defense pathways and responses to environmental stimuli

Interestingly, in addition to DNA damage response, cell cycle regulation, and glucosinolate pathways, our analysis also identified genes of interest reported to be involved in defense and cellular responses to environmental stimuli, such as responses to wounding, bacteria, heat, drought, light stimuli, hypoxia, oxidative stress, and ethylene. The space environment consists not only of radiation and altered gravity, but also contains higher CO_2_, volatile organic compounds (VOCs), water stress, unique microbiomes, and other stressors. Our results demonstrate that even the singular environmental stress of ionizing radiation potentially has a profound impact on the molecular landscape of the plant, and much more work on the impacts of combined stressors will need to be done.

## Conclusions

In conclusion, simulated GCR exposure induces DNA repair mechanisms in a dose-dependent manner and may have a broad impact on plant metabolism that could impact plant growth in space. However, in a deep space radiation scenario, GCR generate a constant extremely low dose rate background radiation field. There are many open questions on how plants respond to constant chronic radiation exposure which need to be investigated further. In our study, we identified DNA repair genes involved in DNA repair mechanisms and changes in glucosinolate biosynthesis related pathways. Responses to environmental stimuli in plants may be significantly altered under radiation exposure and therefore impact deep space long-duration fresh food production as well as space agriculture.

## Data availability statement

The datasets presented in this study can be found in online repositories. The names of the repository/repositories and accession number(s) can be found below: NASA GeneLab data repository, an open-access resource, https://doi.org/10.26030/rxc2-1v61.

## Author contributions

AD: Formal analysis, Methodology, Visualization, Writing – original draft, Writing – review & editing. AM: Formal analysis, Methodology, Visualization, Writing – original draft, Writing – review & editing. BR: Data curation, Formal analysis, Methodology, Writing – review & editing. JR: Investigation, Methodology, Writing – review & editing. SR: Writing – review & editing. SN: Writing – review & editing. HL: Funding acquisition, Writing – review & editing. MC: Writing – review & editing. YZ: Conceptualization, Data curation, Formal analysis, Funding acquisition, Investigation, Methodology, Project administration, Supervision, Visualization, Writing – original draft, Writing – review & editing.
