# Supplementary material for: Simulated galactic cosmic ray exposure activates dose-dependent DNA repair response and down regulates glucosinolate pathways in arabidopsis seedlings
Source: Front Plant Sci. 2023 Dec 14;14:1284529. doi: 10.3389/fpls.2023.1284529 (PMC10757676; doi:10.3389/fpls.2023.1284529)
Supplement: Supplementary file 1 [file Table_1.docx]

Supplementary Material

Simulated Galactic Cosmic Ray Exposure Activates Dose-Dependent DNA Repair Response and Downregulates Glucosinolate Pathway in Arabidopsis Seedlings

**Anirudha R. Dixit^1*†^, Alexander D. Meyers^2†^, Brian Richardson^3†^ , Jeffery T. Richards^1^, Stephanie E. Richards^4^, Srujana Neelam^5^, Howard G. Levine^6^, Mark J. Cameron^3^, and Ye Zhang^6*^**

***Correspondence:**

Ye Zhang

ye.zhang-1@nasa.gov

Anirudha Dixit

anirudha.dixit@nasa.gov

**Supplemental Table 1:** DEGs common to 40 and 80 cGy exposures at FDR *≤* 0.1

| Locus Identifier | Gene Model Description | Primary Gene Symbol | logFC (40 cGy) | logFC (80 cGy) |
| --- | --- | --- | --- | --- |
| AT1G16400 | Encodes cytochrome P450 CYP79F2. | CYTOCHROME P450 | -0.7195 | -0.5278 |
| AT1G23800 | Encodes a mitochondrial aldehyde dehydrogenase; nuclear gene for mitochondrial product. | ALDEHYDE DEHYDROGENASE 2B7 (ALDH2B7) | -0.5636 | -0.5431 |
| AT1G33760 | Encodes a member of the DREB subfamily A-4 of ERF/AP2 transcription factor family. | ETHYLENE RESPONSE FACTOR022 (ERF022) | 1.1699 | 1.554 |
| AT1G73330 | Encodes a plant-specific protease inhibitor-like protein whose transcript level in root disappears in response to progressive drought stress. | DROUGHT-REPRESSED 4 (DR4) | -0.5699 | -0.5687 |
| AT2G30360 | Encodes a SOS2-like protein kinase that is a member of the CBL-interacting protein kinase family. | SOS3-INTERACTING PROTEIN 4 (SIP4) | 0.6396 | 0.9015 |
| AT2G31320 | Encodes a poly (ADP-ribose) polymerase. | POLY(ADP-RIBOSE) POLYMERASE 1 (PARP1) | 0.4780 | 0.6116 |
| AT3G07800 | Encodes a thymidine kinase that salvages DNA precursors. | THYMIDINE KINASE 1A (TK1A) | 0.7731 | 1.3737 |
| AT3G22890 | encodes ATP sulfurylase | ATP SULFURYLASE 1 (APS1) | -0.4290 | -0.3811 |
| AT3G27060 | Encodes one of the 3 ribonucleotide reductase (RNR) small subunit genes. | TSO MEANING 'UGLY' IN CHINESE 2 (TSO2) | 0.7168 | 1.3197 |
| AT4G02390 | Encodes a DNA dependent nuclear poly (ADP-ribose) polymerase (E.C.2.4.2.30) | POLY(ADP-RIBOSE) POLYMERASE 2 (PARP2) | 1.6252 | 2.4284 |
| AT4G04610 | Encodes a protein disulfide isomerase-like (PDIL) protein | APS REDUCTASE 1 (APR1) | -0.6368 | -0.6341 |
| AT5G03780 | Encodes a protein whose sequence is similar to human telomere proteins. | TRF-LIKE 10 (TRFL10) | 0.6901 | 1.1647 |
| AT5G20850 | Encodes a homolog of yeast RAD51. | (RAD51) | 1.1731 | 1.5355 |
| AT5G23010 | Encodes a methylthioalkylmalate synthase | METHYLTHIOALKYLMALATE SYNTHASE 1 (MAM1) | -0.8451 | -0.8017 |
| AT5G26620 | Hypothetical protein;(source: Araport11) |  | 5.5205 | 5.0462 |
| AT5G48720 | Encodes XRI1 (X-ray induced 1). | X-RAY INDUCED TRANSCRIPT 1 (XRI1) | 0.7195 | 1.3728 |
| AT5G49480 | AtCP1 encodes a novel Ca2+-binding protein | CA2+-BINDING PROTEIN 1 (CP1) | 0.4313 | 0.65491 |

**Supplemental Table 2:** Genes exclusively up-regulated due to 40 cGy exposure at FDR *≤* 0.1.

| Locus Identifier | Gene Model Description | Primary Gene Symbol | logFC | FDR |
| --- | --- | --- | --- | --- |
| AT1G62770 | PMEI9 pectin methyleseterase inhibitor. | (PMEI9) | 0.32832 | 0.03635 |
| AT2G23130 | Lysine-rich arabinogalactan-protein (AGP) | ARABINOGALACTAN PROTEIN 17 (AGP17) | 0.49197 | 0.07923 |
| AT2G42170 | Actin family protein |  | 0.41630 | 0.05520 |
| AT3G28635 | hypothetical protein |  | 5.41971 | 0.04051 |
| AT3G51600 | Predicted to encode a PR (pathogenesis-related) protein | LIPID TRANSFER PROTEIN 5 (LTP5) | 0.38191 | 0.06271 |
| AT3G62750 | Encodes a putative beta glucosidase. |  | 0.37792 | 0.05520 |
| AT4G04570 | Encodes a cysteine-rich receptor-like protein kinase. | CYSTEINE-RICH RLK (RECEPTOR-LIKE PROTEIN KINASE) 40 (CRK40) | 0.42529 | 0.09197 |
| AT4G08040 | Encodes an aminotransferase that belongs to ACC synthase gene family structurally | 1-AMINOCYCLOPROPANE-1-CARBOXYLATE SYNTHASE 11 (ACS11) | 0.75789 | 0.08586 |
| AT4G13495 | Other RNA |  | 0.44700 | 0.06766 |
| AT4G34071 | Natural antisense transcript overlaps with AT4G34070 |  | 6.09130 | 0.06746 |
| AT5G12050 | rho GTPase-activating protein | BIG GRAIN 1 (BG1) | 0.49092 | 0.06244 |
| ATMG00670 | transmembrane protein | (ORF275) | 0.60486 | 0.06916 |

**Supplemental Table 3:** Genes exclusively down-regulated due to 40 cGy exposure at FDR *≤* 0.1. Asterisk (*) indicates genes reported to be involved in defense and cellular responses to environmental stimuli.

| Locus Identifier | Gene Model Description | Primary Gene Symbol | logFC | FDR |
| --- | --- | --- | --- | --- |
| AT1G11580^*^ | Pectin methylesterase involved in root growth, plant-pathogen interactions. | PECTIN METHYL ESTERASE INHIBITOR-PECTIN METHYLESTERASE 18 (PMEI-PME18) | -0.7063 | 0.0954 |
| AT1G18880 | Encodes a low-affinity plasma membrane nitrate transporter expressed in the companion cells of root phloem. | NRT1/ PTR FAMILY 2.9 (NPF2.9) | -0.5748 | 0.0087 |
| AT1G21440 | Phosphoenolpyruvate carboxylase family protein |  | -0.6039 | 0.0340 |
| AT1G24100^*^ | Encodes a UDP-glucose: thiohydroximate S-glucosyltransferase, involved in glucosinolate biosynthesis | UDP-GLUCOSYL TRANSFERASE 74B1 (UGT74B1) | -0.4774 | 0.0675 |
| AT1G31710^*^ | Copper amine oxidase family protein | COPPER AMINE OXIDASE ALPHA 3 (CuAOalpha3) | -0.5121 | 0.0815 |
| AT1G44800 | Encodes Siliques Are Red 1 (SIAR1). Functions as a bidirectional amino acid transporter that is crucial for the amino acid homeostasis of siliques. Member of nodulin MtN21-like transporter family. | SILIQUES ARE RED 1 (SIAR1) | -0.6413 | 0.0122 |
| AT1G60260 | beta glucosidase 5 | BETA GLUCOSIDASE 5 (BGLU5) | -0.8551 | 0.0674 |
| AT1G60989^*^ | Encodes a member of a family of small, secreted, cysteine rich proteins with sequence similarity to SCR (S locus cysteine-rich protein). | SCR-LIKE 7 (SCRL7) | -0.5108 | 0.0675 |
| AT1G65860 | Belongs to the flavin-monooxygenase (FMO) family, encodes a glucosinolate S-oxygenase that catalyzes the conversion of methyl thioalkyl glucosinolates to methylsulfinylalkyl glucosinolates | FLAVIN-MONOOXYGENASE GLUCOSINOLATE S-OXYGENASE 1 (FMO GS-OX1) | -1.0069 | 0.0859 |
| AT1G70810 | Calcium-dependent lipid-binding (CaLB domain) family protein | C2-DOMAIN ABA-RELATED7 (CAR7) | -0.7862 | 0.0920 |
| AT1G77885 | hypothetical protein |  | -0.6512 | 0.0792 |
| AT1G78370^*^ | Encodes glutathione transferase belonging to the tau class of GSTs. Naming convention according to Wagner et al. (2002). | GLUTATHIONE S-TRANSFERASE TAU 20 (GSTU20) | -0.8465 | 0.0675 |
| AT1G80080^*^ | Encodes a transmembrane leucine-repeat containing receptor-like protein that is expressed in proliferative post protodermal cells. | TOO MANY MOUTHS (TMM) | -0.8021 | 0.0675 |
| AT1G80870 | Protein kinase superfamily protein |  | -0.6454 | 0.0920 |
| AT2G05440 | GLYCINE RICH PROTEIN 9 | GLYCINE RICH PROTEIN 9 (GRP9) | -1.0555 | 0.0460 |
| AT2G21560 | nucleolar-like protein |  | -0.3226 | 0.0876 |
| AT2G25140^*^ | Encodes ClpB4, which belongs to the Casein lytic proteinase/heat shock protein 100 (Clp/Hsp100) family. | CASEIN LYTIC PROTEINASE B4 (CLPB4) | -0.4092 | 0.0552 |
| AT2G30860^*^ | Encodes glutathione transferase belonging to the phi class of GSTs. Naming convention according to Wagner et al. (2002). | GLUTATHIONE S-TRANSFERASE PHI 9 (GSTF9) | -0.4874 | 0.0559 |
| AT2G37460 | nodulin MtN21-like transporter family protein | USUALLY MULTIPLE ACIDS MOVE IN AND OUT TRANSPORTERS 12 (UMAMIT12) | -0.5982 | 0.0624 |
| AT2G40900 | Encodes a plasma membrane-localized amino acid transporter likely involved in amino acid export in the developing seed. | USUALLY MULTIPLE ACIDS MOVE IN AND OUT TRANSPORTERS 11 (UMAMIT11) | -0.6431 | 0.0675 |
| AT2G46650 | member of Cytochromes b5 | CYTOCHROME B5 ISOFORM C (CB5-C) | -0.8748 | 0.0920 |
| AT2G47540^*^ | Pollen Ole e 1 allergen and extensin family protein |  | -1.8137 | 0.0078 |
| AT3G05330 | Encodes a protein with moderate sequence similarity to the maize microtubule-binding protein TANGLED1. | TANGLED (ATN) | -0.6931 | 0.0630 |
| AT3G14570 | Encodes a protein similar to callose synthase | GLUCAN SYNTHASE-LIKE 4 (GSL04) | -0.5873 | 0.0624 |
| AT3G19710 | Belongs to the branched-chain amino acid aminotransferase gene family. Encodes a methionine-oxo-acid transaminase. | BRANCHED-CHAIN AMINOTRANSFERASE4 (BCAT4) | -1.0389 | 0.0027 |
| AT3G43190^*^ | Encodes a protein with sucrose synthase activity (SUS4). | SUCROSE SYNTHASE 4 (SUS4) | -1.7784 | 0.0231 |
| AT3G48770 | ATP/DNA binding protein |  | -1.3864 | 0.0805 |
| AT3G54600 | Glyoxalase I with low activity. | (DJ1F) | -0.7151 | 0.0078 |
| AT3G54640^*^ | Catalyzes the conversion of indole-3-glycerolphosphate to indole, the penultimate reaction in the biosynthesis of tryptophan. | TRYPTOPHAN SYNTHASE ALPHA CHAIN (TSA1) | -0.4384 | 0.0907 |
| AT3G55500 | Expansin-like protein. | EXPANSIN A16 (EXPA16) | -0.7421 | 0.0907 |
| AT4G12030 | Required for the biosynthesis of methionine-derived glucosinolates. Involved in the transport of 2-keto acids between chloroplasts and the cytosol. | BILE ACID TRANSPORTER 5 (BAT5) | -0.7330 | 0.0606 |
| AT4G14480 | Encodes a putative Ser/Thr protein kinase | BLUE LIGHT SIGNALING1 (BLUS1) | -0.8728 | 0.0675 |
| AT4G29080^*^ | phytochrome-associated protein 2 (PAP2) | PHYTOCHROME-ASSOCIATED PROTEIN 2 (PAP2) | -0.4757 | 0.0859 |
| AT4G30450 | glycine-rich protein |  | -0.7750 | 0.0675 |
| AT4G31730 | Glutamine dumper1 is a putative transmembrane protein. It is involved in glutamine secretion. | GLUTAMINE DUMPER 1 (GDU1) | -0.6516 | 0.0393 |
| AT5G07460^*^ | Ubiquitous enzyme that repairs oxidatively damaged proteins. | PEPTIDEMETHIONINE SULFOXIDE REDUCTASE 2 (PMSR2) | -0.7634 | 0.0678 |
| AT5G14200 | The AtIMD1 is one out of 3 genes encoding the enzyme 3-isopropylmalate dehydrogenase involved in leucine biosynthesis in Arabidopsis. | ISOPROPYLMALATE DEHYDROGENASE 1 (IMD1) | -0.6566 | 0.0503 |
| AT5G23020 | Methylthioalkymalate synthase-like. | 2-ISOPROPYLMALATE SYNTHASE 2 (IMS2) | -0.9240 | 0.0002 |
| AT5G54390 | Encodes a 3'-phosphoadenosine-5'-phosphate (PAP) phosphatase that is sensitive to physiological concentrations of Na+. | HAL2-LIKE (HL) | -0.3460 | 0.0552 |

**Supplemental Table 4:** Genes exclusively up-regulated due to 80 cGy exposure at FDR *≤* 0.1.

| Locus ID | Gene Model Description | Gene Symbol | logFC | FDR |
| --- | --- | --- | --- | --- |
| AT1G08260 | Similar to POL2A, DNA polymerase epsilon catalytic subunit | TILTED 1 (TIL1) | 0.5148 | 0.0965 |
| AT1G49980 | DNA/RNA polymerases superfamily protein |  | 0.4665 | 0.0658 |
| AT2G07741 | ATPase, F0 complex, subunit A protein |  | 8.2299 | 0.0074 |
| AT2G18193 | P-loop containing nucleoside triphosphate hydrolases superfamily protein |  | 1.6557 | 0.0000 |
| AT2G31320 | Encodes a poly (ADP-ribose) polymerase. | POLY(ADP-RIBOSE) POLYMERASE 1 (PARP1) | 0.6146 | 0.0121 |
| AT2G45460 | SMAD/FHA domain-containing protein |  | 0.6695 | 0.0009 |
| AT3G07650 | Belongs to the CO (CONSTANS) gene family. | CONSTANS-LIKE 9 (COL9) | 0.3496 | 0.0591 |
| AT3G58270 | phospholipase-like protein (PEARLI 4) with TRAF-like domain protein |  | 0.7436 | 0.0741 |
| AT4G21070 | Encodes AtBRCA1, an ortholog of the human breast cancer susceptibility gene 1. | BREAST CANCER SUSCEPTIBILITY1 (BRCA1) | 0.9369 | 0.0124 |
| AT5G07610 | F-box family protein |  | 3.6394 | 0.0000 |
| AT5G40840 | Cohesion family protein SYN2 (SYN2). | (SYN2) | 0.7061 | 0.0499 |
| AT5G48020 | 2-oxoglutarate (2OG) and Fe (II)-dependent oxygenase superfamily protein |  | 0.3602 | 0.0391 |
| AT5G60250 | zinc finger (C3HC4-type RING finger) family protein |  | 1.0396 | 0.0052 |
| AT5G61380 | Pseudo response regulator involved in the generation of circadian rhythms. | TIMING OF CAB EXPRESSION 1 (TOC1) | 0.3341 | 0.0900 |
| AT5G66130 | Encodes a homolog to yeast RAD17. | RADIATION SENSITIVE 17 (ATRAD17) | 1.0802 | 0.0911 |

**Supplemental Table 5:** Genes exclusively down-regulated due to 80 cGy exposure at FDR *≤* 0.1.

| Locus Identifier | Gene Model Description | Primary Gene Symbol | logFC | FDR |
| --- | --- | --- | --- | --- |
| AT1G12530 | Regulator of SNC1 and RPP4 mediated immunity. Interacts with methyltransferase ATXR7. | MODIFIER OF SNC1 9 (MOS9) | -0.7379 | 0.0390 |
| AT1G54960 | member of MEKK subfamily | NPK1-RELATED PROTEIN KINASE 2 (NP2) | -1.2628 | 0.0496 |
| AT2G18360 | alpha/beta-Hydrolases superfamily protein |  | -1.3364 | 0.0680 |
| AT4G04830 | methionine sulfoxide reductase B5 | METHIONINE SULFOXIDE REDUCTASE B5 (MSRB5) | -0.7739 | 0.0111 |
| AT5G06280 | hypothetical protein |  | -0.4229 | 0.0961 |
| AT5G43180 | transmembrane protein, putative (Protein of unknown function, DUF599) |  | -1.1653 | 0.0397 |
| AT5G44400 | FAD-binding Berberine family protein | (ATBBE26) | -0.8291 | 0.0961 |
| ATMG00500 | hypothetical protein | (ORF141) | -3.2657 | 0.0013 |
